# Optimal Design of Anger Camera for Bremsstrahlung Imaging: Monte Carlo Evaluation

**DOI:** 10.3389/fonc.2014.00149

**Published:** 2014-06-13

**Authors:** Stephan Walrand, Michel Hesse, Randy Wojcik, Renaud Lhommel, François Jamar

**Affiliations:** ^1^Department of Nuclear Medicine, Molecular Imaging, Radiotherapy and Oncology Unit (MIRO), IECR, Université Catholique de Louvain, Brussels, Belgium; ^2^Ray Visions Inc., Yorktown, VA, USA

**Keywords:** yttrium-90, bremsstrahlung, tomography, Monte Carlo, radioembolization, dosimetry

## Abstract

A conventional Anger camera is not adapted to bremsstrahlung imaging and, as a result, even using a reduced energy acquisition window, geometric x-rays represent <15% of the recorded events. This increases noise, limits the contrast, and reduces the quantification accuracy. Monte Carlo (MC) simulations of energy spectra showed that a camera based on a 30-mm-thick BGO crystal and equipped with a high energy pinhole collimator is well-adapted to bremsstrahlung imaging. The total scatter contamination is reduced by a factor 10 versus a conventional NaI camera equipped with a high energy parallel hole collimator enabling acquisition using an extended energy window ranging from 50 to 350 keV. By using the recorded event energy in the reconstruction method, shorter acquisition time and reduced orbit range will be usable allowing the design of a simplified mobile gantry. This is more convenient for use in a busy catheterization room. After injecting a safe activity, a fast single photon emission computed tomography could be performed without moving the catheter tip in order to assess the liver dosimetry and estimate the additional safe activity that could still be injected. Further long running time MC simulations of realistic acquisitions will allow assessing the quantification capability of such system. Simultaneously, a dedicated bremsstrahlung prototype camera reusing PMT–BGO blocks coming from a retired PET system is currently under design for further evaluation.

## Introduction

Recent studies proved the tumor response and the hepatic toxicity correlated to their respective absorbed doses in liver ^90^Y radioembolization (1–5). This strongly supports the need to individually determine the maximum safe activity that can be injected in order to optimize the patient outcome. Unfortunately, in the mean time, accumulating evidence showed that pre-therapy ^99m^Tc-macroagregated (MAA) distribution is not reliable in predicting the ^90^Y microsphere distribution on a patient by patient basis (6, 7). Thus, a way to quantitatively assess the ^90^Y microsphere distribution in the catheterization room is highly valuable.

Quantitatively imaging ^90^Y with an Anger camera is one of the most challenging topics in nuclear medicine. The bremsstrahlung x-rays are spread along a continuous spectrum extending up to the maximal beta energy emission, i.e., 2.3 MeV. The maximum energy usable by a camera using a mechanical collimator being about 0.5 MeV, acquisitions are contaminated by high energy x-rays scattered down into the acquisition energy window. The major problematic effects are: (1) the scattering inside the patient body, (2) the collimator scattering and penetration, (3) the lead fluorescence around 80 keV in the collimator, (4) the back-scattering from the light guide, the photo-multiplier tubes (PMTs), electronic boards, and lead housing of the camera. As a result, contrary to gamma emission imaging where about 60% of the recorded events are geometric γ-rays, geometric x-rays in bremsstrahlung imaging represent <15% of the recorded events (8). Numerous methods have been proposed to address these issues [see (9, 10) for an extensive bibliography].

State of the art bremsstrahlung single photon emission computed tomography (SPECT) implements collimator–detector table point spread function (PSF) pre-calculated by Monte Carlo (MC) in the iterative reconstruction process (11, 12). These methods show improved quantification in phantoms (12) and in patient studies as well (13). However, in nuclear medicine imaging it is always profitable to improve the hardware performance in order to select the right events, rather than to correct for contaminating events afterward. Indeed, this last solution inevitably results in a higher noise level regarding the statistical nature of geometric and contaminating x-rays. Other choices than parallel hole collimators have been recently considered.

Van Holen et al. (14) proposed the use of a rotating slat collimator that has a much higher geometric efficiency than a parallel hole collimator. The relative importance of septal penetration is reduced, resulting in a better contrast to noise ratio. Note that, regarding only the geometric x-rays, the high geometric efficiency improvement of the rotating slat collimator is counterbalanced by less information provided about the x-rays coming direction. Also, the high energy x-rays fraction crossing the crystal, and afterward back scattered down in the energy acquisition window, is not reduced.

Walrand et al. (15) has used a medium energy pinhole (MEPH) collimator in bremsstrahlung SPECT. Collimator penetration–scattering can only occur on the small area of the tungsten nose insert in contrast with parallel hole collimator where the scattering occurs on the inner walls of each hole. Evaluation of a cold and hot spheres phantom showed that MEPH SPECT provided quantification accuracy similar to that of time of flight–positron emission tomography (TOF–PET), but with significantly less noise. Helical MEPH SPECTs of a liver radioembolization phantom were also acquired and showed that reproducibly accurate activity quantification can be obtained within a 3 min acquisition time.

In this paper, we evaluate by MC simulations the optimal design of an Anger camera–collimator system for bremsstrahlung imaging. We discuss the reasons why the use of the recorded event energy allowed by such optimal system should allow shortening the acquisition time and reducing the camera orbit range in SPECT. These improvements are well-adapted to fast liver dosimetry assessment in the catheterization room. Last, we introduce our current prototype project evaluation.

## MC Code

Simulations were performed in the vGate 2.1 environment (16) running Gate 6.2 and Geant4.9.5.p01 (17). Geant4 (GEometry ANd Tracking) is a toolkit for the simulation of the passage of particles through matter initially developed in order to model particle and nuclear physics experiments. GATE (Geant4 Application for Tomographic Emission) is an advanced opensource software developed by the international OpenGATE collaboration and dedicated to numerical simulations in medical imaging and radiotherapy using Geant4. In order to profit from recent corrections, the source files handling the atom de-excitations and fluorescence emissions of Geant4.9.5.p01 were replaced by those of Geant4.9.6.p02. Accordingly, the call to these processes was updated in the LiverMore model source file. Afterward, Geant4 and Gate were successively rebuilt. Simulations were performed using the LiverMore model for the photoelectric and for the Compton processes. The relative yields of the Pb fluorescence peaks checked in the simulations were in agreement with those reported in the G4EMLOW (Geant4 ElectroMagnetic Low energy) data file.

## MC Validation

In order to validate our MC simulations, a ^90^Y point source surrounded by 1 cm perpex was acquired using a 1/2^″^-thick NaI (Sodium Iodine) GE400AC camera (General Electric, Milwaukee, WI, USA): our only camera having a MEPH collimator. The GE400AC was successively equipped with a low energy high resolution (LEHR), a medium energy general purpose (MEGP), a high energy general purpose (HEGP), and an MEPH collimator. The distance between the source and the collimators aperture was 10 cm.

**Figure 1:**
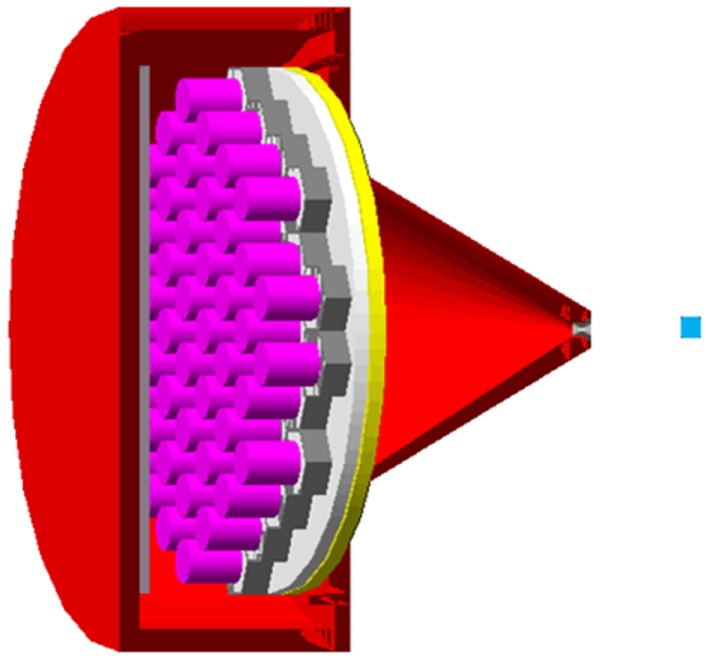
**Gate modeling of the GE400AC equipped with the MEPH collimator**. From right to left: blue: perpex surrounding the ^90^Y point source, gray: 6 mm-aperture tungsten insert, red: lead housing, yellow: 1/2^″^-thick NaI crystal, white: 12 mm-thick pyrex light guide, gray: 0.2 mm-thick hexagonal mu-metal shielding, purple: cathode–dynode cascade (PMTs pyrex housing was set no visible), gray: 1 cm-thick perpex modeling the electronics.

A retired PMT from the GE400AC was taken apart for measurement of the housing pyrex thickness, of the cathode–dynodes cascade dimensions and weight. The PMTs were modeled by hexagonal–cylindrical pyrex tubes, each one containing a copper medium of volume and of effective density derived from the previous measurements (Figure 1). The hexagonal bases of the PMTs were surrounded by 0.2 mm-thick mu-metal. A 12 mm-thick pyrex light guide was set between the PMTs and the crystal. Last, a 1 cm-thick perpex plate was set behind the PMTs in order to model the electronics.

**Figure 2:**
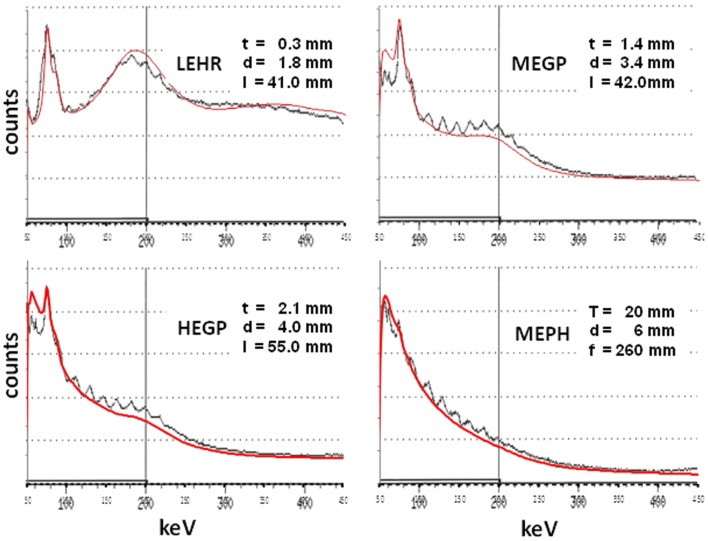
**Comparison between the acquisitions (black curves) and the MC simulations (red curves) for a ^90^Y point source surrounded by 1 cm of perpex and set 10 cm far from a LEHR, an MEGP, a HEGP, and an MEPH collimator, all mounted on a 1/2^″^-thick NaI GE400AC camera**. *t* is the septal thickness, *d* the hole diameter, *l* the hole length. For MEPH, *T* is the front housing thickness, and *f* the focal length. Note the absence of lead fluorescence peak for the MEPH collimator. The oscillations observed in the acquisitions from 100 to 220 keV (17 keV-period) were not explained.

Figure 2 shows the comparison between the acquisitions and the MC simulations. Overall, the total counts shapes were well-reproduced for a wide variety of parallel hole collimators, ranging from a low energy to a high energy parallel hole collimator, and including an MEPH collimator as well. The lead fluorescence peaks were well-reproduced for all the parallel hole collimators and also its absence when using the pinhole collimator. This absence arises from the fact that the x-rays cannot reach the inner side of the MEPH housing (15). In contrary, using a parallel hole collimator, x-rays hit the inner side of all the collimator holes producing fluorescence photons, which are able to reach the crystal.

Some small discrepancies were observed below the lead fluorescence peaks for the MEGP and HEGP collimators, while the back-scatter peak was a little bit overestimated for the LEHR collimator and a little bit underestimated for the other ones. One possible explanation is that these collimators were produced at different epochs and by different companies (our GE400AC has a 26 years long history). Manufactures often use lead alloys containing some percentage of tin (up to 15% and unknown to us) in order to improve the casting properties. Last, for obscure reasons, the GE400AC energy spectra displayed structured oscillations between 100 and 220 keV.

## Optimal Collimator Design: MC Simulations

There are three adverse effects, which occur in the collimator: the lead K-shell fluorescence, the penetration, and the scattering (15).

Lead fluorescence in a pinhole collimator can be reduced by using a bi-conical insert made in tungsten for the cone facing the activity, but made in tin for the cone facing the crystal in order to attenuate the tungsten and lead fluorescence x-rays (see second last section). Tin and antinomy are the highest atomic number elements (*Z* = 50 and 51, respectively) with high density (≈7 kg/dm^3^) having their K-shell fluorescence below 50 keV [≈30 keV (18)]. Additionally, the inner side of the lead front plate of the pinhole collimator can be covered by a 2 mm-thick tin layer in order to attenuate the fluorescence produced by the small amount of high energy x-rays crossing the lead plate. Except for the GE400AC, all the pinhole collimators were modeled with these two features.

**Figure 3:**
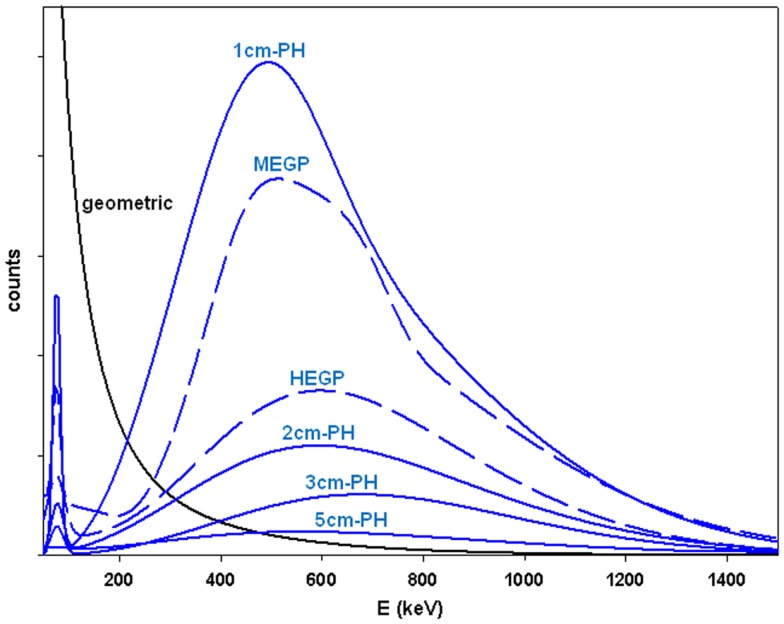
**MC simulations of the x-rays counts passing through the collimator material (blue curves) for a ^90^Y point source surrounded by 1 cm perpex and set 10 cm far from the collimator**. The curves were rescaled in order to get the same geometric x-rays component (black curve) for all the collimators. Septa thickness is 1.4 and 3.2 mm, hole diameter is 3.4 and 4.0 mm, hole length is 42 and 40 mm, for the MEGP and HEGP collimator, respectively. The length in centimeters is the thickness of the lead front plate of the pinhole (PH) collimator. The aperture diameter is 8 mm and the focal length is 15 cm.

Figure 3 shows the penetration and scatter component of various collimators, i.e., the x-rays passing through the collimator material without any interaction or with Compton scattering. Most of these x-rays are above the usual energy acquisition window. However, some of them will leave some energy within the acquisition window by Compton scattering in the crystal and some will be back scattered down into the energy acquisition window by the camera compartment. This dramatically hampers the image contrast and the quantification accuracy (9).

The penetration and scatter component is reduced when using a HEGP collimator in place of the MEGP one. However for a similar spatial resolution, a conventional MEPH collimator (2 cm-housing wall thickness) already displays a lower penetration and scatter component. Furthermore, the septa thickness of a parallel hole collimator cannot indefinitely be increased without hampering the spatial resolution and sensitivity. In contrary, the housing wall thickness of a pinhole collimator can be increased without any drawback on the collimator performances, the holding weight capacity of the gantry being the only limitation. A 45 cm × 25 cm front lead housing wall, a size well-adapted to whole-liver imaging, weights about 64 kg for a 5 cm-thickness. Further increasing of the thickness only resulted in small improvement (data not shown).

## Optimal Crystal Choice

The main drawback of a conventional Anger camera for bremsstrahlung imaging is the back-scattering by the pyrex light guide and PMTs of the high energy x-rays passing through the crystal (9).

Heard et al. (8) assessed by MC simulation the lead fluorescence, the geometric x-rays, and the camera back-scatter x-rays originating from a ^90^Y point source in water detected by a 3/8^″^-thick NaI Anger camera equipped with a medium energy parallel hole collimator. Below 90 keV, due to the limited crystal energy resolution, the geometric x-rays are significantly contaminated by lead fluorescence and above 90 keV the geometric x-rays represent only a decreasing fraction of the camera back-scatter x-rays. As a result, the energy window optimizing the contrast is often constrained to (100, 150) keV (i.e., between 100 and 150 keV) (8), which limits the sensitivity.

**Figure 4:**
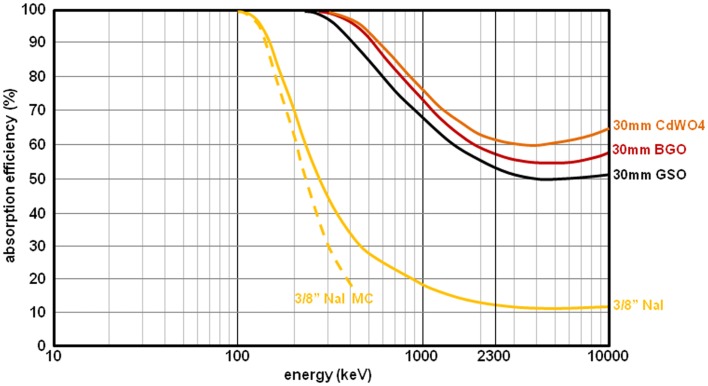
**Absorption efficiency of 3/8^″^-thick NaI (yellow curve), of 30 mm-thick GSO (black curve), of 30 mm-thick BGO (red curve), and of 30 mm-thick CdWO4 (brown curve)**. The solid curves were derived from Ref. (19). The dash curve (MC simulation) was extracted from Ref. (20).

The camera back-scatter contamination can be reduced by using a thick crystal having a high photoelectric efficiency. This solution also has the additional benefit of improving the geometric x-rays sensitivity. Gd_2_SiO_5_ (GSO, gadolinium oxyorthosilicate) and Bi_4_Ge_3_O_12_ (BGO, bismuth germanate) are two good candidates. BGO has an absorption efficiency a little better than that of GSO: 90 and 84% for 30 mm-thickness at 511 keV, respectively, both being much better than that of a conventional 3/8^″^-thick NaI crystal, i.e., ≈15% (Figure 4). GSO has the benefit to produce a little bit more light than BGO, i.e., 12.5 and 9 photons/keV, respectively, providing a better energy resolution. BGO has the drawback of a strong temperature dependence of the light production, i.e., −1.2%/K. This can induce some shift of the energy spectrum. However, when the purpose is to acquire bremsstrahlung x-rays within a large energy acquisition window, energy resolution and temperature dependence have only a marginal impact.

The best crystal for a dedicated bremsstrahlung camera is CdWO_4_ (cadmium tungstate) that has an absorption efficiency a little better than BGO (Figure 4) and a light yield slightly better than GSO, i.e., 13.5 photons/keV. However, the primary light decay of CdWO4 is slow (14 μs). As a result, the field of view should be split in independent crystal–PMT–application specific integrated circuit (ASIC) units in order to handle the count rate required in liver radioembolization.

## BGO Camera Design: MC Simulations

**Figure 5:**
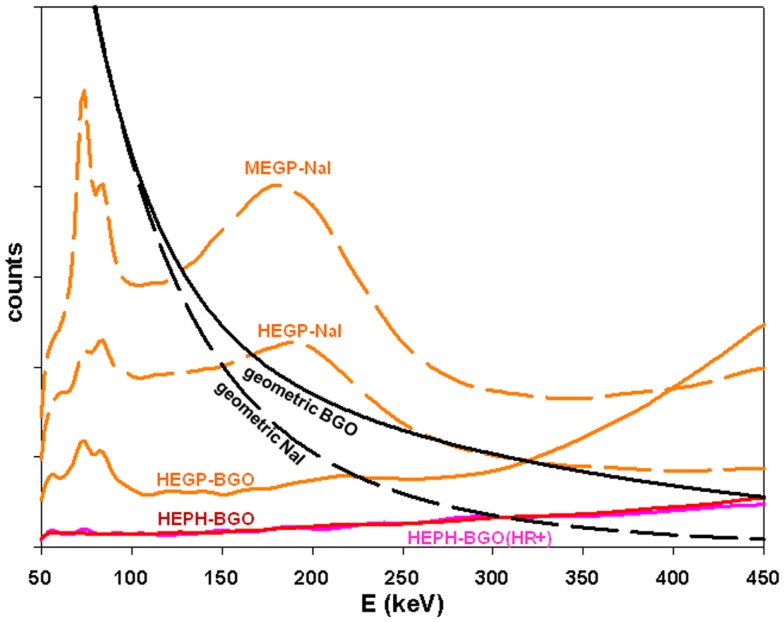
**Comparison of the total scatter components (colored curves) with the geometric x-rays (black curves) obtained by MC simulations for a ^90^Y point source surrounded by 1 cm of perpex and set 10 cm in front of the collimator**. Septa thickness is 1.4 and 3.2 mm, hole diameter is 3.4 and 4.0 mm, hole length is 42 and 40 mm, for the MEGP and HEGP collimator, respectively. Front wall thickness of 5 cm, aperture diameter of 8 mm, focal length of 15 cm for the HEPH collimator. 45 cm × 25 cm crystal: 3/8^″^-thick NaI and 30 mm-thick BGO. The purple curve corresponds to the BGO prototype (see second last section). The BGO to NaI geometric ratio is 4.8 at 400 keV, which is in line with the 4.9 predicted by previous MC simulations (Figure 4).

Figure 5 shows the total scatter component, i.e., collimator scatter-penetration + camera backscattering, using MEGP, HEGP, and high energy pinhole (HEPH) collimator mounted on a 3/8^″^-thick NaI and on a 30 mm-thick BGO camera, both with a crystal size of 45 cm × 25 cm. Simulations were performed with a 6.6 cm-thick slab of 66% density Pyrex modeling, the camera back compartment (8). The ^90^Y point source surrounded by 1 cm of perpex was set 10 cm far from the collimator. With a HEGP collimator, the scatter component crossed the geometric x-rays around 150 and 320 keV using NaI and BGO, respectively. Using a 5 cm-thick pinhole collimator, this last intersection was shifted to 450 keV.

## Potential Benefit Provided by the Extended Acquisition Energy Window

In addition to improve the sensitivity, the extension of the acquisition energy window could provide a much more important benefit.

**Figure 6:**
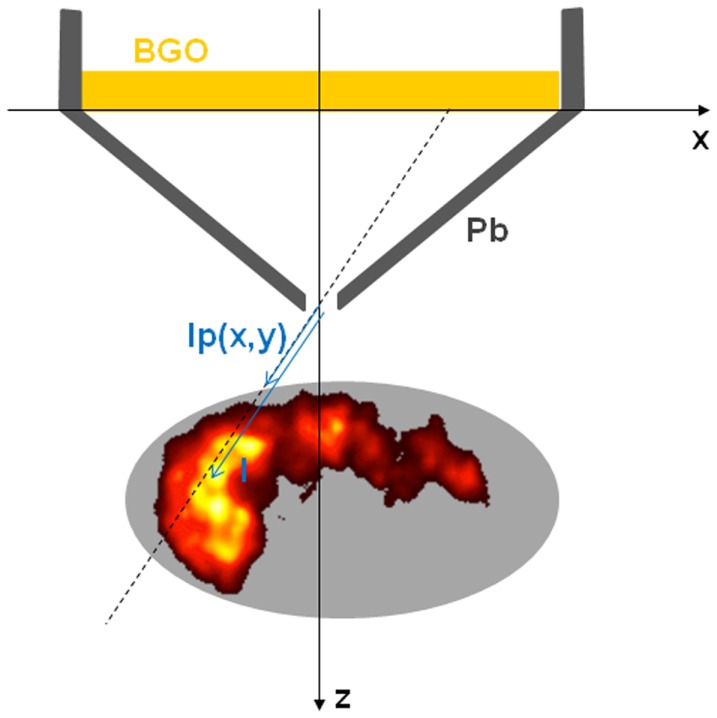
**Coordinates convention for the pinhole collimator line of response**.

Let’s assume that we have an ideal camera–collimator system, i.e., no fluorescence, no scattering, no septal or housing wall penetration, a perfect energy, and a perfect spatial resolution, and that the patient tissue in the liver slice is similar to water (which is a reasonable assumption in liver imaging). Then, neglecting the scattered x-rays, the count rate *c*(*x, y, E*) recorded at the energy *E* in the pixel (*x, y*) of the camera stationary above the patient is:

(1) $$\begin{array}{llll} c\left(x,y,E\right) & =\int_{0}^{\infty }a\left(E\right)A\left(x\prime \left(l\right),y\prime \left(l\right),z\prime \left(l\right)\right)G\left(x\prime \left(l\right),y\prime \left(l\right),z\prime \left(l\right)\right)\; & & \;\\ & \;e^{-\mu \left(E\right)\left(l-l_{p}\left(x,y\right)\right)}dl \end{array}$$

where *A*(*x*′, *y*′, *z*′) is the ^90^Y activity, *G*(*x*′, *y*′, *z*′) the geometric efficiency of the collimator, *a*(*E*) is the bremsstrahlung x-rays yields in water at energy *E*, μ(*E*) the x-ray attenuation coefficient of water at energy *E*. [*x*′(*l*), *y*′(*l*), *z*′(*l*)] is the parametric equation of the collimator line of response (Figure 6), i.e., for a parallel hole collimator:

(2) x′(l)=x;y′(l)=y;z′(l)=l

and for a pinhole collimator:

(3) $$\begin{array}{llll} x\prime \left(l\right) & =-\frac{l}{\sqrt{F^{2}+x^{2}+y^{2}}}x;y\prime \left(l\right)=-\frac{l}{\sqrt{F2+x2+y2}}y;\; & & \;\\ z\prime \left(l\right) & =F+\frac{lF}{\sqrt{F^{2}+x^{2}+y^{2}}} \end{array}$$

*l* is the distance on the line of response from the crystal for the parallel hole collimator and from the aperture for the pinhole collimator. *lp*(*x, y*) is the *l* value on the intersection of the line of response with the patient surface facing the collimator.

Dividing both sides of Eq. (1) by $a\left(E\right)e^{\mu \left(E\right)l_{p}\left(x,y\right)}$, we get:

(4) $$\begin{array}{llll} & C\left(x,y,E\right)\; & & \;\\ & \;=\int_{0}^{\infty }A\left(x\prime \left(l\right),y\prime \left(l\right),z\prime \left(l\right)\right)G\left(x\prime \left(l\right),y\prime \left(l\right),z\prime \left(l\right)\right)e-\mu \text{(}E\text{)l}dl \end{array}$$

where

(5) $$C\left(x,y,E\right)=c\left(x,y,E\right)e^{-\mu \left(E\right)l_{p}\left(x,y\right)}∕a\left(E\right)$$

μ(E) for water is a bijective function ranging from 0 (*E* = ∞) to ∞ (*E* = 0) (18). Inverting this function gives:

(6) $$\hat{C}\left(x,y,\mu \right)=\int_{0}^{\infty }\bar{A}\left(l\right)e^{-\mu l}dl$$

where:

(7) $$\begin{array}{ll} \hat{C}\left(x,y,\mu \right) & =C\left(x,y,E\left(\mu \right)\right) \end{array}$$

(8) $$\begin{array}{ll} \bar{A}\left(l\right) & =A\left(x\prime \left(l\right),y\prime \left(l\right),z\prime \left(l\right)\right)G\left(x\prime \left(l\right),y\prime \left(l\right),z\left(l\right)\right) \end{array}$$

Equation 6 is just the Laplace transform, the inversion of which arises in various engineering and physics problems, e.g., such as electronic circuits analysis or heat conduction modeling (21). The inversion is unique except for an additional arbitrary null-function *N*(*l*), i.e., $\int_{0}^{l}N\left(l\prime \right)dl\prime =0\forall l>0$, which is known as Lerch’s theorem. For the engineers and physicists, *N*(*l*) may almost be taken as zero (21). Thus, in theory, Eq. (6) allows us to compute *A*(*x*′, *y*′, *z*′) via Eq. (8). Equation 6 is the equivalent of the Radon transform where the energy *E*, via the attenuation, plays the role of the rotation angle φ in conventional SPECT.

However, the inversion of the Laplace transform appears to be a much more ill posed problem than conventional SPECT (22), especially when the Laplace transform is known, or is measured, only on the real positive axis (23, 24). Numerous inversion methods and regularization schemes exist, and the choice of the optimal couple requires a detailed analysis of the problem to be solved, supported by numerous simulations.

In real situation, similarly to conventional SPECT, the acquisition will be affected by the limited energy and spatial resolution of the camera, and by the Compton scattering occurring inside the camera–collimator and inside the patient as well, which can be simulated only by MC methods. Simulation of a single acquisition requires some months of CPU time on a 20 cores computer. A faster MC algorithm dedicated to bremsstrahlung is under development.

Likely, regarding the noise inherent to radioactive decay imaging, Eq. (6) alone will not be sufficient to obtain a reliable activity reconstruction. However, similarly to the time of flight (TOF) introduction in PET (25, 26), the utilization of the recorded event energy in the reconstruction process should allow a reduction of the acquisition time and on the angular orbit range, resulting in a simplified camera mobile gantry. This is important for the use in a busy catheterization room. After injecting a safe activity, a fast SPECT, without moving the catheter tip, could be performed in order to assess the liver dosimetry and estimate the additional safe activity that could still be injected.

## BGO Bremsstrahlung Dedicated Camera Prototype

**Figure 7:**
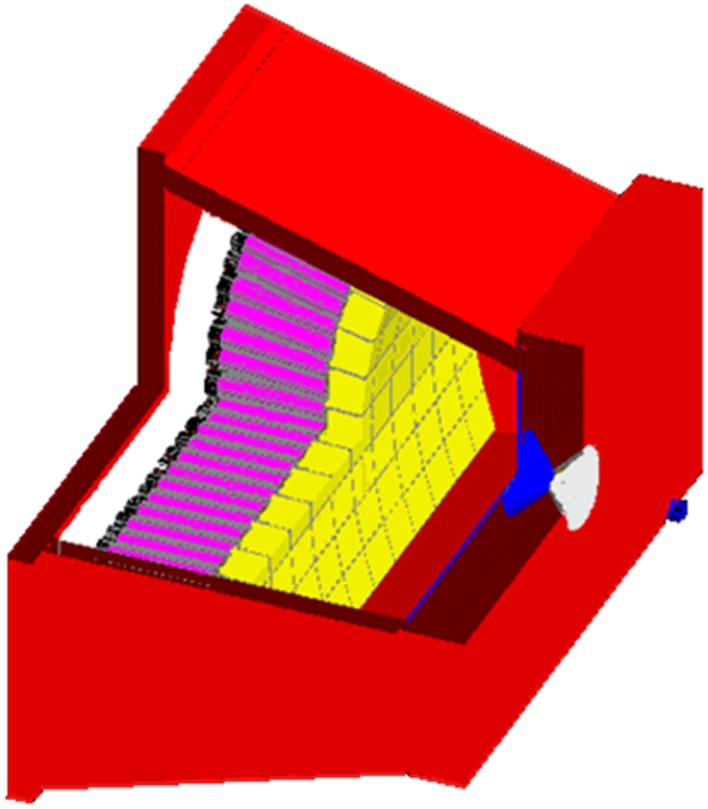
**Gate modeling of the prototype made of 7 × 11 BGO-blocks coming from a retired Exact HR + PET and equipped with a pinhole collimator (5 cm-thick lead front plate, 8 mm-aperture diameter)**. From right to left: blue: 1 cm-thick perpex surrounding the ^90^Y point source, red: lead housing, white: tungsten outer conical insert, blue: tin inner conical insert and 2 mm-thick layer, yellow: 30 mm-thick BG, gray: mu-metal, purple: cathode–dynodes cascade (PMT pyrex housing walls were set no visible), black: PMT plastic sockets, white: perpex modeling the electronics.

In order to assess the performance of a dedicated bremsstrahlung camera–collimator and of the benefit of the extended acquisition energy window, we plan to build a camera prototype by reusing PMT–BGO blocks of a retired Exact HR + PET (CTI, Knoxville, TN, USA). The BGO block thickness is 30 mm with an overall front size of 35 mm × 38 mm divided into 8 × 8 pixels. The prototype will be made of 11 rows, each having 7 PMT–BGO blocks set along an arc (Figure 7) in order to avoid gaps between the front sides of the BGO blocks that have a trapezoidal shape. The detector FOV will be 25 cm × 43 cm. The detector arc shape is useful when using the camera with a pinhole by reducing the parallax error in the rows direction due to the depth of interaction.

Similarly that for the GE400AC modeling, a BGO-block was disassembled and one PMT was taken apart to measure the pyrex and cathode–dynode cascade dimensions and weight. An accurate modeling of the prototype equipped with a pinhole collimator (5 cm-thick lead front plate, 8 mm-aperture tungsten insert) was performed (Figure 7). The MC simulations showed that an energy acquisition window extended to (50, 350) keV could be used (Figure 5). Access to a super computer will be required in order to simulate a complete acquisition.

In order to account for the different interactions of the x-rays in the collimator and camera, the PSF (*x, y, z, E*) of a ^90^Y point source will be measured for different water depths *z*, and used in the reconstruction process. Since the BGO light yield is temperature dependent (−1.2%/K), use of the recorded event energy with BGO requires measuring these PSF with the camera being in thermal equilibrium. The energy spectrum of each block for a ^99m^Tc source will also be recorded in this thermal equilibrium. A thin temperature probe will be set in contact with the two central BGO blocks to assess the thermal equilibrium. Before each patient acquisition, the energy spectrum of the blocks using a ^99m^Tc source will again be recorded and used to rescale the energy measurement of the bremsstrahlung x-rays during the liver acquisition in order to account for difference in temperatures from 1 day to another one.

## Conclusion

Monte Carlo simulations prove a significant reduction of the total scatter component when using a 30 mm-thick BGO camera equipped with a HEPH collimator (i.e., 5 cm-thick). With this camera, the total scatter component cross the geometric x-rays counts around 450 keV rather than below 150 keV when using a conventional 3/8^″^-NaI camera equipped with a parallel hole collimator.

In addition to improve conventional SPECT, mathematics shows that such novel performances should allow the development of a simplified mobile gantry, which is more convenient for use in a busy catheterization room during the liver radioembolization. Liver dosimetry assessment will be possible without moving the catheter tip in order to estimate the additional safe activity that could still be injected. The development of a faster MC code dedicated to bremsstrahlung imaging is under way in order to predict the feasible camera orbit range reduction.

## Author Contributions

All authors contributed to the conception, the revision, the approval and agreed with the work. Stephan Walrand initiated the work. Stephan Walrand and Michel Hesse made the MC simulations. Randy Wojcik handled the camera acquisition features. François Jamar and Renaud Lhommel handled the camera design regarding its use during liver radioembolization.

## Conflict of Interest Statement

BGO prototype camera development and the article processing fee are funded by Sirtex Medical Limited, Sydney, NSW, Australia

## References

[1] Flamen P Vanderlinden B Delatte P Ghanem G Ameye L Van Den Eynde M Multimodality imaging can predict the metabolic response of unresectable colorectal liver metastases to radioembolization therapy with Yttrium-90 labeled resin microspheres. Phys Med Biol (2008) 53:6591–603 10.1088/0031-9155/53/22/019 18978442

[2] Walrand S Lhommel R Goffette P Van den Eynde M Pauwels S Jamar F Hemoglobin level significantly impacts the tumor cell survival fraction in humans after internal radiotherapy. EJNMMI Res (2012) 2:20 10.1186/2191-219X-2-20 22608186 PMC3413597

[3] Strigari L Sciuto R Rea S Carpanese L Pizzi G Soriani A Efficacy and toxicity related to treatment of hepatocellular carcinoma with 90Y-SIR spheres: radiobiologic considerations. J Nucl Med (2010) 51:1377–85 10.2967/jnumed.110.075861 20720056

[4] Chiesa C Mira M Maccauro M Romito R Spreafico C Sposito C A dosimetric treatment planning strategy in radioembolization of hepatocarcinoma with 90Y glass microspheres. Q J Nucl Med Mol Imaging (2012) 56:503–8 23358402

[5] Walrand S Hesse M Jamar F Lhommel R A hepatic dose-toxicity model opening the way toward individualized radioembolization planning. J Nucl Med (2014). 10.2967/jnumed.113.135301 24904111

[6] Jiang M Fischman A Nowakowski FS Heiba S Zhang Z Knesaurek K Segmental perfusion differences on paired Tc-99m macroaggregated albumin (MAA) hepatic perfusion imaging and yttrium-90 (Y-90) bremsstrahlung imaging studies in SIR-sphere radioembolization: associations with angiography. J Nucl Med Radiat Ther (2012) 3:1 10.4172/2155-9619.1000122

[7] Chiesa C Maccauro M Romito R Spreafico C Pellizzari S Negri A Need, feasibility and convenience of dosimetric treatment planning in liver selective internal radiation therapy with (90)Y microspheres: the experience of the National Tumor Institute of Milan. Q J Nucl Med Mol Imaging (2011) 55: 168–97 21386789

[8] Heard S Flux GD Guy MJ Ott RJ Monte Carlo simulation of 90Y Bremsstrahlung imaging. IEEE Nucl Sci Symp Conf Rec (2004) 6:3579–83 10.1109/NSSMIC.2004.1466658

[9] Walrand S Flux GD Konijnenberg MW Valkema R Krenning EP Lhommel R Dosimetry of yttrium-labelled radiopharmaceuticals for internal therapy: 86Y or 90Y imaging? Eur J Nucl Med Mol Imaging (2011) 38:S57–68 10.1007/s00259-011-1771-7 21484382

[10] Walrand S Bremsstrahlung SPECT/CT. In: Ahmadzadehfar H Biersack HJ, editors. Clinical Applications of SPECT-CT. Berlin: Springer (1998). p. 271–80

[11] Minarik D Sjogreen Gleisner K Ljungberg M Evaluation of quantitative (90) Y SPECT based on experimental phantom studies. Phys Med Biol (2008) 53:5689–703 10.1088/0031-9155/53/20/008 18812648

[12] Rong X Du Y Ljungberg M Rault E Vandenberghe S Frey EC Development and evaluation of an improved quantitative 90Y bremsstrahlung SPECT method. Med Phys (2012) 39:2346–58 10.1118/1.3700174 22559605 PMC3338590

[13] Minarik D Sjögreen-Gleisner K Linden O Winga K Tennvall J Strand S-E 90Y bremsstrahlung imaging for absorbed-dose assessment in high-dose radioimmunotherapy. J Nucl Med (2010) 51:1974–8 10.2967/jnumed.110.079897 21078799

[14] Van Holen R Staelens S Vandenberghe S SPECT imaging of high energy isotopes and isotopes with high energy contaminants with rotating slat collimators. Med Phys (2009) 36:4257–67 10.1118/1.3177312 19810500

[15] Walrand S Hesse S Demonceau G Pauwels S Jamar F Yttrium-90 labeled microspheres tracking during liver selective internal radiotherapy by bremsstrahlung pinhole SPECT: feasibility study and evaluation in an abdominal phantom. EJNMMI Res (2011) 1:32 10.1186/2191-219X-1-32 22214246 PMC3377914

[16] Jan S Santin G Strul D Staelens S Assié K Autret D GATE: a simulation toolkit for PET and SPECT. Phys Med Biol (2004) 49:4543–61 10.1088/0031-9155/49/19/007 15552416 PMC3267383

[17] Agostinelli S Allison J Amako K Apostolakis J Araujo H Arce P Geant4 – a simulation toolkit. Nuclear Instruments and Methods in Physics Research Section A: Accelerators, Spectrometers, Detectors and Associated Equipment (2003) 506(3):250–303 10.1016/S0168-9002(03)01368-8

[18] Hubbell JH Seltzer SM Tables of X-Ray Mass Attenuation Coefficients and Mass Energy-Absorption Coefficients from 1 keV to 20 MeV for Elements Z = 1 to 92 and 48 Additional Substances of Dosimetric Interest. National Institute of Standards and Technology (2009). Available from: http://www.nist.gov/pml/data/xraycoef/index.cfm

[19] Saint-Gobain Ceramics & Plastics, Inc. Efficiency Calculations for Selected Scintillators (2004). Available from: http://www.lip.pt/~luis/docs/bicron-eff.pdf

[20] Suzuki S Detection efficiency of NaI (Tl) crystals and loss of position resolution caused by photon interactions in the crystals in y-cameras. Int J Appl Radiat Isot (1982) 33:411–4 10.1016/0020-708X(82)90038-2 7107036

[21] Arfken GB Weber HJ Mathematical Methods for Physicists. Oxford: Elsevier Academic Press (2004).

[22] Bertero M Boccacci P Introduction to Inverse Problems in Imaging. Bristol: Institute of Physics Publishing (1998).

[23] Cohen AM Numerical Methods for Laplace Transform Inversion. New York: Springer Science+Business Media (2007).

[24] Gzyl H Tagliani A Milev M Laplace transform inversion on the real line is truly ill-conditioned. Appl Math Comput (2013) 219:9805–9 10.1016/j.amc.2013.03.112

[25] Conti M Effect of randoms on signal-to-noise ratio in TOF PET. IEEE Trans Nucl Sci (2006) 53:1188–93 10.1109/TNS.2006.875066

[26] Vandenberghe S Lemahieu I System characteristics of simulated limited angle TOF PET. Nucl Instrum Methods Phys Res A (2007) 71:480–3 10.1016/j.nima.2006.10.139

